# Short Daily versus Conventional Hemodialysis for Hypertensive Patients: A Randomized Cross-Over Study

**DOI:** 10.1371/journal.pone.0097135

**Published:** 2014-05-29

**Authors:** Deborah L. Zimmerman, Marcel Ruzicka, Paul Hebert, Dean Fergusson, Rhian M. Touyz, Kevin D. Burns

**Affiliations:** 1 Associate Professor of Medicine, Division of Nephrology, Ottawa Hospital, Kidney Research Centre of the Ottawa Hospital Research Institute, University of Ottawa, Ottawa, Ontario, Canada; 2 Professor of Medicine, Surgery, Anesthesia and Epidemiology, University of Ottawa; Senior Scientist, Clinical Epidemiology, Ottawa Hospital Research Institute, Ottawa, Ontario, Canada; 3 Associate Professor, Departments of Medicine, Surgery, Epidemiology and Community Medicine, Senior Scientist and Director, Clinical Epidemiology Program, Ottawa Hospital Research Institute, Ottawa, Ontario, Canada; 4 Institute of Cardiovascular and Medical Sciences, BHF Glasgow Cardiovascular Research Centre, University of Glasgow, Glasgow, United Kingdom; 5 Professor of Medicine, Division of Nephrology, Senior Scientist, Kidney Research Centre of the Ottawa Hospital Research Institute, University of Ottawa, Ottawa, Ontario, Canada; Rouen University Hospital, France

## Abstract

**Background:**

Treatment of end stage renal disease patients with short daily hemodialysis has been associated with an improvement in blood pressure. It is unclear from these studies if anti-hypertensive management had been optimized prior to starting short daily hemodialysis. Also, the potential mechanism(s) of blood pressure improvement remain to be fully elucidated.

**Study Design, Setting and Participants:**

We undertook a randomized cross-over trial in adult hypertensive patients with ESRD treated with conventional hemodialysis to determine: 1) if short-daily hemodialysis is associated with a reduction in systolic blood pressure after a 3-month blood pressure optimization period and; 2) the potential mechanism(s) of blood pressure reduction. Blood pressure was measured using Canadian Hypertension Education Program guidelines. Extracellular fluid volume (ECFV) was assessed with bioimpedance. Serum catecholamines were used to assess the sympathetic nervous system. Interleukin-6 (IL-6) and thiobarbituric acid reactive substances (T-BARS) were used as markers of inflammation and oxidative stress respectively.

**Results:**

After a 3-month run-in phase in which systolic blood pressure improved, there was no significant difference in pre-dialysis systolic pressure between short-daily and conventional hemodialysis (p = 0.39). However, similar blood pressures were achieved on fewer anti-hypertensive medications with short daily hemodialysis compared to conventional hemodialysis (p = 0.01). Short daily hemodialysis, compared to conventional hemodialysis, was not associated with a difference in dry weight or ECFV (p = 0.77). Sympathetic nervous system activity as assessed by plasma epinephrine (p = 1.0) and norepinephrine (p = 0.52) was also not different. Markers of inflammation (p = 0.42) and oxidative stress (p = 0.83) were also similar between the two treatment arms.

**Conclusions:**

Patients treated with short daily, compared to conventional hemodialysis, have similar blood pressure control on fewer anti-hypertensive medications. The mechanism(s) by which short daily hemodialysis allows for decreased anti-hypertensive medication use remains unclear but effects on sodium balance and changes in peripheral vascular resistance require further study.

**Trial Registration:**

ClinicalTrials.gov NCT00759967

## Introduction

More than 50% of the patients with end stage renal disease (ESRD) die from cardiovascular disease, a risk 10–20 times greater than the general population [1], [2]. Of the potentially modifiable cardiovascular risk factors, greater than 80% of patients with ESRD have hypertension; 70% of whom are poorly controlled using conventional therapy [3]. An expanded extracellular fluid volume (ECFV) and an increase in peripheral vascular resistance (PVR) due to hemodynamic/trophic effects of increased sympathetic nerve activity and inflammation are frequently quoted mechanisms contributing to hypertension in ESRD [4]. Common to these processes is increased bioavailability of reactive oxygen species [5].

The intermittent nature of conventional hemodialysis treatments (4 hours, 3 days/week) results in the majority of patients having a fluctuating ECFV that likely contributes to the activation of neurohormonal pathways [6]. However, daily hemodialysis therapy including short daily (2 hours, 6 days/week) and nocturnal (6–8 hours, 5–6 days/week) improves or even normalizes blood pressure [6]–[12]. Short daily hemodialysis reportedly improves blood pressure secondary to a reduction in ECFV [6], [9] whereas nocturnal hemodialysis appears to be associated with a reduction in PVR [9]–[11]. This suggests that different mechanisms of blood pressure reduction may vary in different patient groups. In a study by Katzarski et al, patients who were normotensive and dialyzed for 7–8 hours, 3 days/week were compared to normotensive and hypertensive patients whose treatment time was 3–5 hours [13]. There was no overall difference in the ECFV and inferior vena cava diameter between the two normotensive groups, which were lower than the hypertensive patients. However, 24% of the patients treated with long dialysis appeared to have an expanded ECFV such that the authors hypothesized that normotension in these patients might be explained by the removal of a vasoactive hormone. Their results are supported by a small randomized controlled trial of blood pressure control in 21 ESRD patients in which 7 patients had their dialysis time increased by 2 hours, 6 patients had their dialysis time increased by 2 hours and dry weight reduced, and 8 patients only had their dry weight reduced [14]. Despite a decrease in dry weight (and presumably ECFV) in only 2 of the groups, systolic blood pressure and use of anti-hypertensive medication use was reduced in all 3 groups.

In the majority of reported studies it is unclear if the patient's antihypertensive management, including achievement of the appropriate dry weight, had been optimized prior to the introduction of more intensive hemodialysis. A limited number of investigations have been undertaken to determine the mechanisms of blood pressure reduction. For this reason, we undertook a randomized cross-over trial in patients with ESRD treated with hemodialysis. Our primary objective was to determine if short-daily hemodialysis, compared to conventional hemodialysis, is associated with a reduction in systolic blood pressure pre-dialysis after an optimization period. Our secondary objective was to determine the potential mechanism(s) of blood pressure reduction by assessing extracellular fluid volume and the sympathetic nervous system. The potential role of inflammation and oxidative stress were added to the study design because of the increasing evidence suggesting that these mediators may have an etiologic role in hypertension [5], [15].

## Methods

### Study Overview

We undertook a 9-month randomized, crossover trial of adult prevalent hypertensive ESRD patients. Paperwork for the trial was submitted to the Ottawa Hospital Research Institute in June 2007 for registration at clinicaltrials.gov as per protocol at the time but due to administrative delays was not released until September 24, 2008 (NCT00759967). The protocol for this trial and supporting CONSORT checklist is available; see Checklist S1 and Protocol S1. The Ottawa Hospital Research Ethics Board approved the study and all amendments. The study was conducted according to the Declaration of Helsinki.

### Run-in Phase

**Figure 1:**
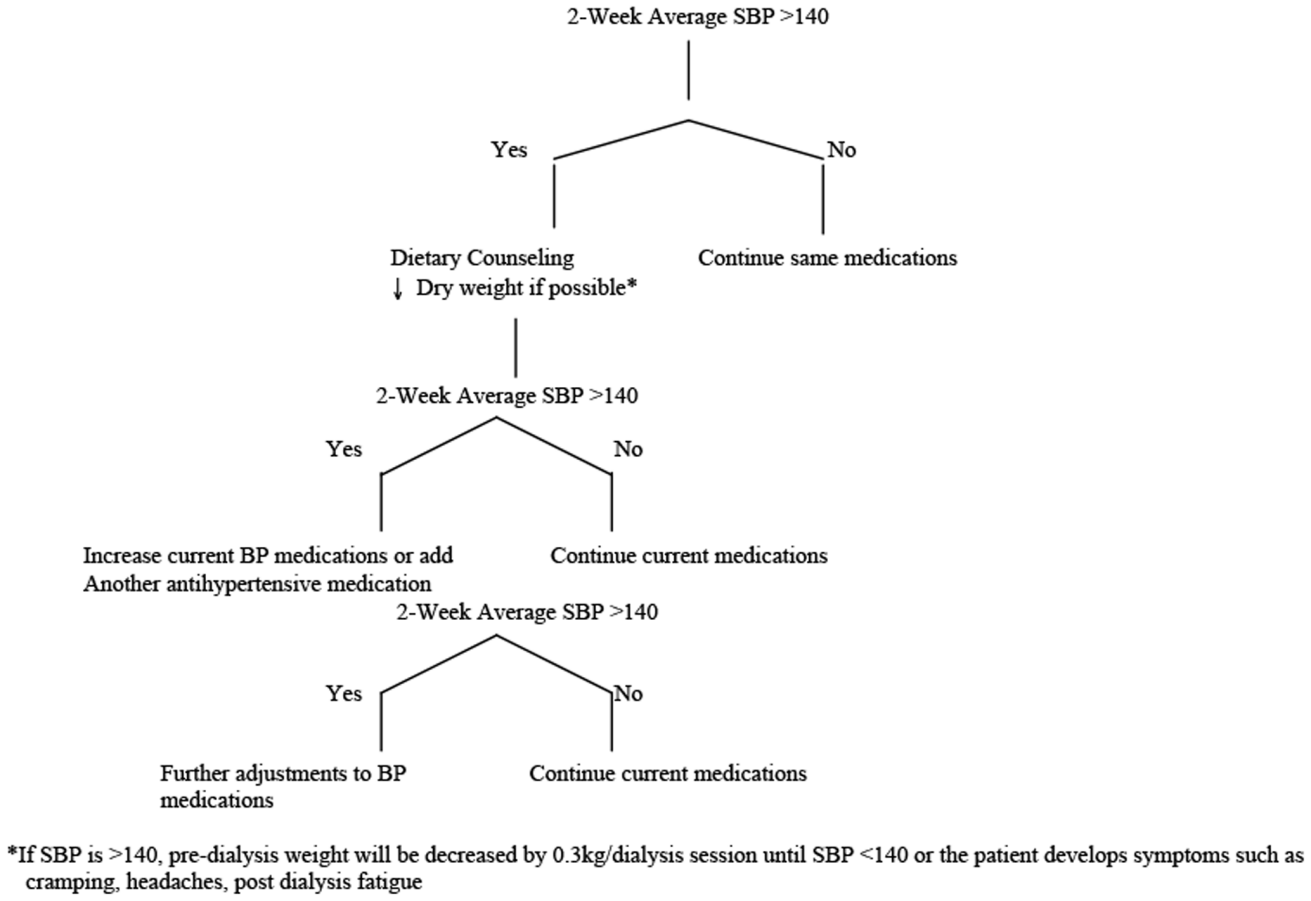
Protocolized Blood Pressure Management Algorithm.

Inclusion criteria included: 1) on hemodialysis for >3 months, 2) any patient on 1 or 2 antihypertensive medications with a pre-dialysis systolic blood pressure of >140 on average over 1 month, 3) any patient on 3 or more antihypertensive medications regardless of pre-dialysis systolic pressure, 4) able to make the time commitment to daily therapy, and 5) able to give informed consent. Patients were excluded if they were expected to switch treatment modality or die within one year or have known dilated cardiomyopathy with an ejection fraction <0.3. Eligible patients provided written informed consent to the study coordinator and then underwent a 3-month run-in phase. All patients were dialyzed with a Fresenius K machine, Optiflux 160 dialyzer, blood pump speed 300–400 mls/min and a dialysate flow of 500 mls/minute. A protocolized blood pressure algorithm was followed to optimize blood pressure control (Figure 1). Briefly, all patients met with the renal dietician. The dietary sodium (<2 grams per day) and fluid restrictions (1L per day) as per unit policy were re-enforced. The dialysate sodium was reduced to 138 mmol/L from 140 mmol/L, the latter being the standard sodium concentration in our Ottawa Hospital hemodialysis units. At each hemodialysis treatment, a pre and post hemodialysis blood pressure was measured using techniques recommended by the Canadian Hypertension Education program. Changes were made to dry weight and/or antihypertensive medications every 2 weeks by the principal investigator if the 2-week average pre-dialysis systolic blood pressure was greater than 140 mmHg. Forty-four hour ambulatory blood pressure was also measured in the last week of the run-in phase. The number, type and strength of antihypertensive medications were recorded at study entry and at the end of each month. Intensity of anti-hypertensive medication use was estimated as multiples of the smallest available dose for that particular medication (eg. amlodipine - smallest available dose 5 mg = 1: 10 mg = 2, 2.5 mg = 0.5). Standard of care monthly laboratory investigations (complete blood count, electrolytes, calcium, phosphate, albumin, creatinine and pre/post hemodialysis urea were collected at baseline and the last month of the run-in phase on a Wednesday or Thursday pre-dialysis.

### Randomization

**Figure 2:**
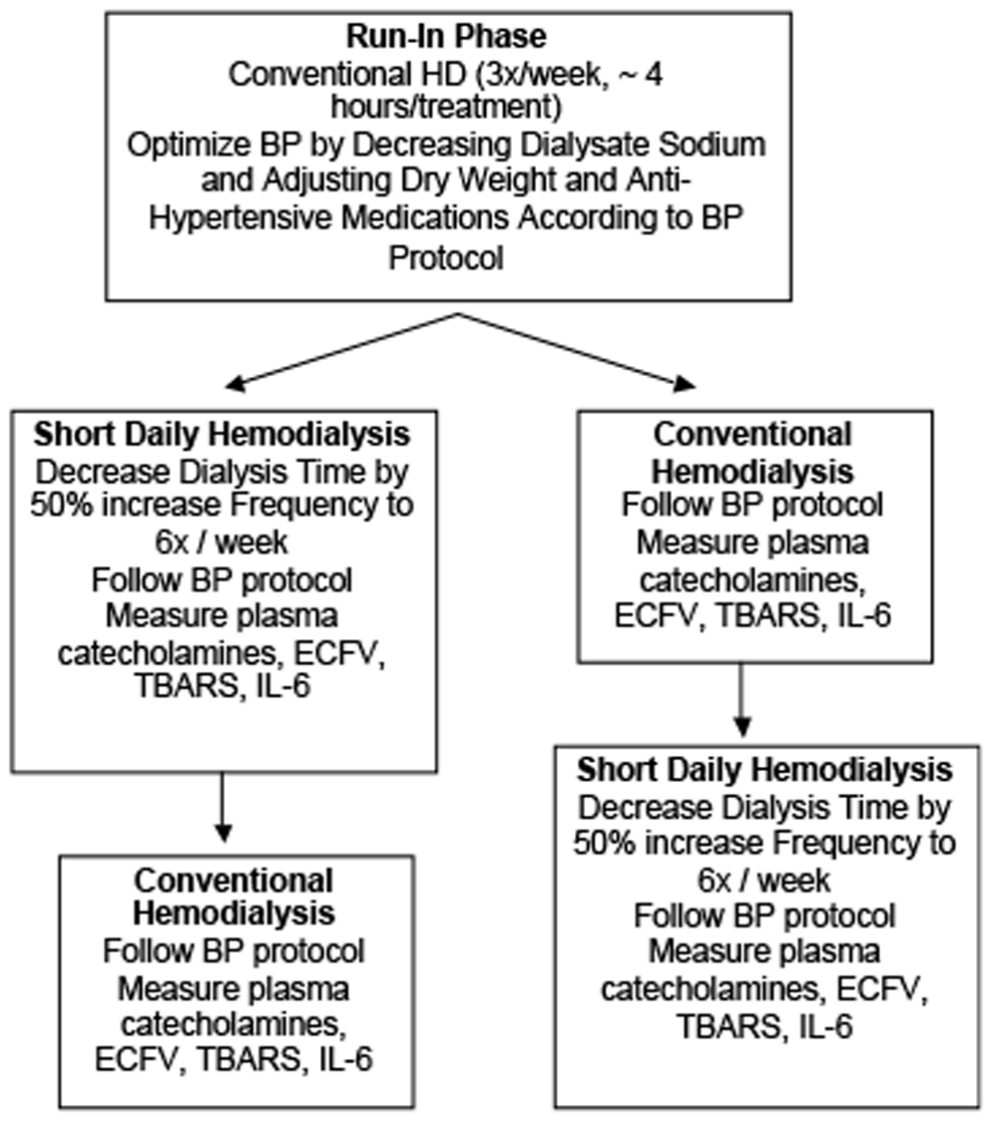
Flow Diagram.

After the 3 month run-in phase, patients were randomized using a computer generated randomization sequence. Each patient completed 3 months of conventional 3 times per week hemodialysis or 6 times per week short daily hemodialysis and then crossed-over to the other treatment arm (Fig. 2). The only differences in the short daily dialysis prescription was to increase the frequency from 3 days to 6 days, decrease the hemodialysis session time by 50% and decrease the dialysate bicarbonate concentration to 32 mmol/L from 36 mmol/L to avoid metabolic alkalosis [16]. The assessment of blood pressure with adjustments to weight and anti-hypertensive medications was the same as for the run-in phase. Standard monthly laboratory investigations were also the same except during short daily hemodialysis, blood was collected on Monday (after a day off).

### Assessment of Extracellular Fluid Volume

Post treatment extracellular fluid volume was measured with the Xitron Hydra (Xitron Technologies, San Diego, CA) using standard techniques. The Hydra is a multifrequency bioimpedance analyzer that allows for differentiation of ECFV and intracellular fluid volume by detecting differences in the conductive properties of cells by measuring resistance (impedance) to electrical current. The technique is reliable in tracking sequential changes in ECFV with a reported coefficient of variation and intra-observer error of 2.5% [13], [17]. All measurements were taken 20 minutes after completion of the hemodialysis treatment, by placing 4 electrodes on the wrist and ankle on the non-fistula side. Yearly calibration of the equipment was performed by the Ottawa Hospital technical department.

### Measurement of Plasma Catecholamines

Blood samples were taken post dialysis with the patient in a quiet dark room prior to the removal of the hemodialysis needles. The plasma samples were prepared immediately and stored at −80C and analyzed within 6 months by high performance liquid chromatography at a commercial laboratory.

### Markers of Inflammation and Oxidative Stress

Plasma interleukin-6 (IL-6) and plasma thiobarbituric acid reactive substances (T-BARS) were used as markers of inflammation and oxidative stress respectively. Plasma was prepared from blood collected in EDTA vacutainer tubes pre-dialysis and stored at −80°C. Plasma levels of IL-6 were measured using a solid-phase sandwich ELISA kit from Biosource International according to manufacturer's instructions. Plasma TBARS levels were measured colorimetrically and values were expressed in nmol/ml malondialdehyde (MDA) equivalents as we previously reported [18], [19].

### Analysis

Based on our previous experience with daily hemofiltration, we calculated that 20 patients would be required to detect a difference of 11 mmHg in systolic blood pressure assuming a standard deviation of 11, a two-sided alpha = 0.05, and a power of 80% [20].

Results are expressed as means and standard deviations or median and interquartile range depending on normality assumptions for continuous data and frequency for categorical data as appropriate. The primary analysis was intention to treat paired t-test of the comparison of the mean pre-dialysis systolic blood pressure values from the last month on conventional hemodialysis and the last month of short daily hemodialysis (last value carried forward for missing blood pressure data). As described by Zoccali et al, this gives an assessment of the pressure load as experienced by the left ventricle [21]. To assess order effect (or period effect) a GEE model with SBP as outcome and treatment and period as predictors, accounting for patient effect in repeated measures was performed.

A paired t-test was also used for weight, ECFV, total ultrafiltration and ultrafiltration rate. Due to the non-normal distribution of the hypertension medication intensity score, serum catecholamines, IL-6 and TBARs, these were compared using the Wilcoxin sign rank test. Linear regression analysis was undertaken to explore the predictors of the intensity of anti-hypertensive medication use after conventional and daily hemodialysis (post-hoc).

## Results

**Figure 3:**
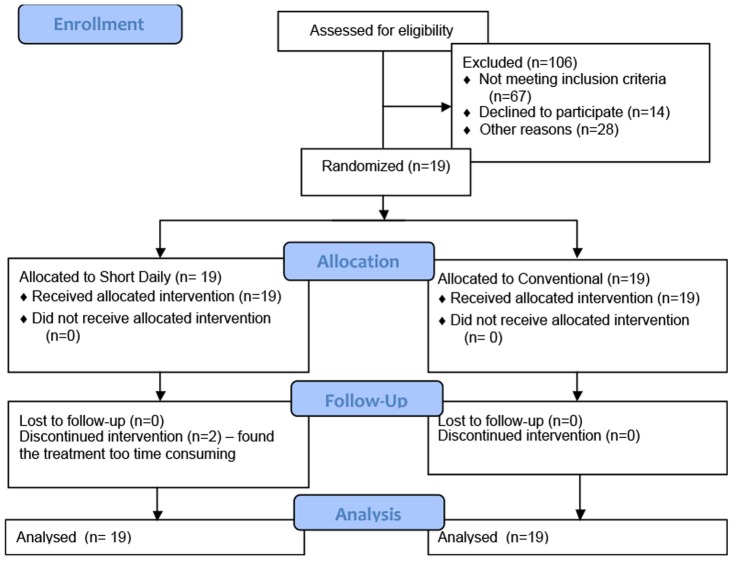
Trial Flow.

Twenty-two patients consented to participate in the study; 3 patients withdrew prior to randomization, 2 patients did not complete the short daily hemodialysis (Figure 3). Therefore data from 19 patients were included in the systolic blood pressure analysis. Patient demographics are depicted in Table 1. The average age of the participating patients was 53 years, 11 patients (58%) had a history of diabetes mellitus and the majority of patients were male.

**Table 1:** Baseline Patient Characteristics.

Patient Characteristics	
Age – Years (SD)	53 (13)
Gender (M:F)	13:6
Dialysis Vintage – Day (SD)	1368 (1670)
Charlson Comorbidity Score (SD)	6 (2)
Etiology of ESRD	DM (7), GN (6), HTN (2), Multiple Myeloma (1), Amyloid (1), Other (2)
History of Diabetes Mellitus (Y/N)	11/8
Access Type (AVF/AVG/CVC)	11/2/6
Dry Weight (kgs)	77.4 (14.3)
Baseline BP (SD)	151 (12)/79 (9)
HTN Medication Intensity Score	5.46 (2.94)

SD – standard deviation, M – Male, F – Female, HTN – hypertension, ESRD – end stage renal disease,

DM – diabetes mellitus, GN – glomerulonephritis (includes IgA, Goodpasture's, Immunofibrillary), Y/N – yes/no,

AVF – arteriovenous fistula, AVG – arteriovenous graft, CVC – central venous catheter, kgs – kilograms.

There was a statistically significant decrease in systolic blood pressure from study entry to the end of the run in phase (151 vs 138 mmHg; p = 0.004) without an overall change in dry weight (77.4 vs 77.1 kgs; p = 0.63) or intensity of antihypertensive medications (5.46 vs 5.16; p = 0.57). Although we did not demonstrate a reduction in average post dialysis body weight at the end of the run-in phase compared to baseline, there was tremendous variability with the weights of some participants being decreased (maximum 8.7 kgs) and the weights of other participants being increased (maximum 5.4 kgs). Similar changes were seen in the anti-hypertensive medication use from baseline to the end of the run-in phase with some patients requiring increased medication while other patients had decreased medication. Overall, there was a decline in the amount of ultrafiltration per treatment from the first month of the run-in phase to the last month of the run-in phase (2.5L vs 2.3L; p = 0.007).

Of the standard biochemical tests throughout the study, serum sodium was 138 mmol/L at baseline and 137 mmol/L at the end of the run-in phase. This change was maintained throughout the study. Serum phosphate declined from 1.86 mmol/L at baseline to 1.61 mmol/L at the end of the run-in phase (p = 0.04). During conventional hemodialysis and short daily hemodialysis after randomization, serum phosphate was 1.82 mmol/L and 1.58 mmol/L respectively (p = 0.25). Weekly Kt/V was similar at the end of the run in phase and 3 months of conventional HD (5.4) but increased (6.6) during short daily HD. No changes were observed in serum potassium, calcium, bicarbonate or albumin throughout the study.

**Figure 4:**
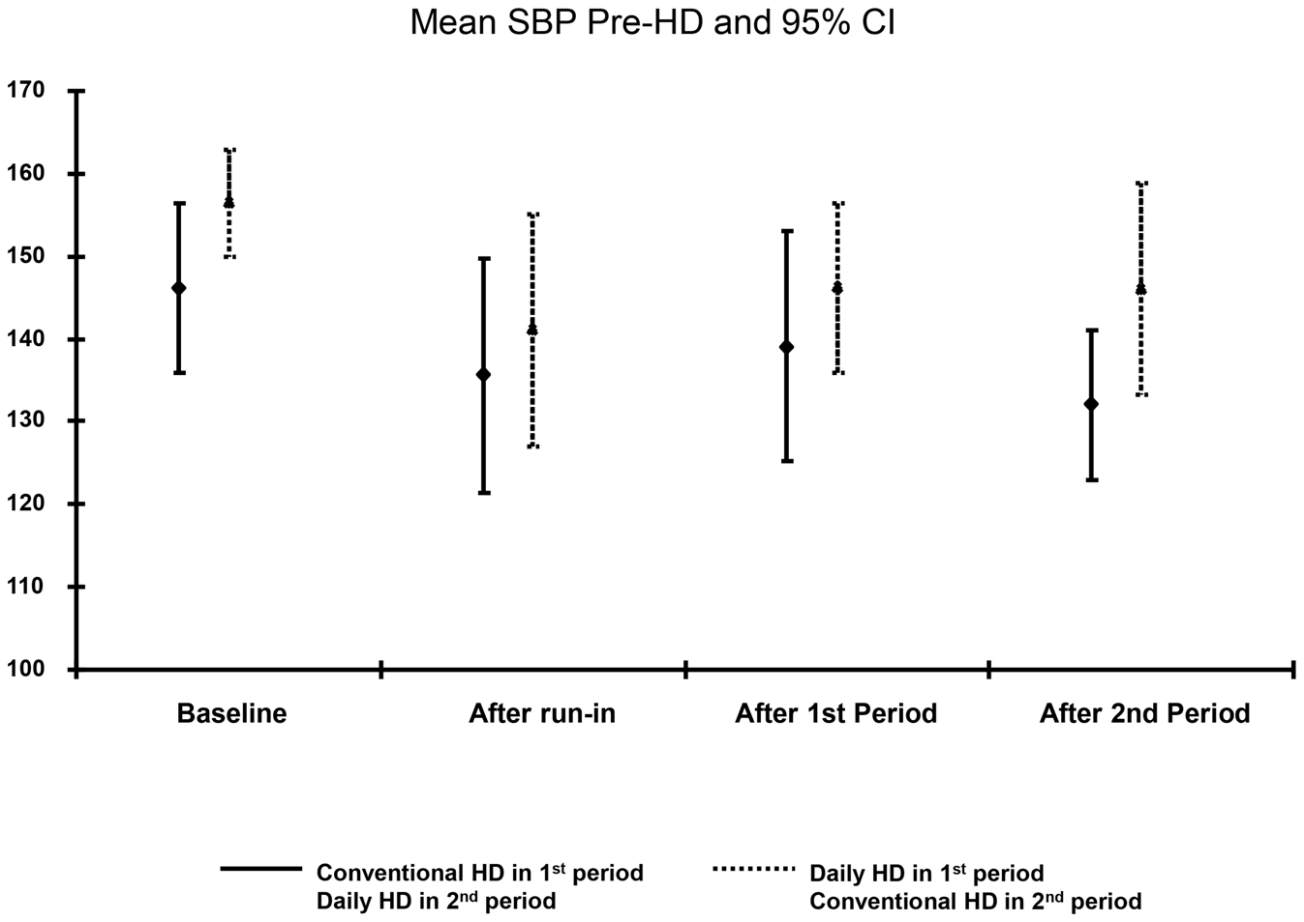
Graph of Period Effects.

Changes in the antihypertensive medication classes are shown in Table 2 at baseline, after the run-in, short daily and conventional hemodialysis phases. After 3 months of short daily, compared to 3 months of conventional hemodialysis, systolic and diastolic blood pressures were not statistically different (p = 0.39, p = 0.56 respectively; Table 3) and there was no period effect (p = 0.40, figure 4). These results are supported by the 44-hour ABPM. However, these similar blood pressures were achieved on a lower intensity of hypertension medication use in the short daily hemodialysis phase compared to conventional hemodialysis phase (p = 0.01, Table 4). There were no significant differences in dry weight, ECFV, plasma epinephrine or nor-epinephrine levels between conventional hemodialysis and short daily hemodialysis (Table 4). There was no difference between IL-6 and TBARS by treatment modality. Plasma catecholamines, ECFV and serum phosphate were not significantly associated with antihypertensive medication intensity score on conventional (p = 0.42, 0.88, 0.09 respectively) or short daily hemodialysis (p = 0.24, 0.22, 0.68 respectively).

**Table 2:** Changes in Classes of Anti-Hypertensive Medications during the Trial.

	Baseline	Run-In	CHD	DHD
Ace-I	10	9	10	8
ARB	8	5	4	3
β-blocker	11	9	6	6
DHCCB	14	13	11	8
α-blocker	5	2	2	1
Central sympatholytic	1	1	1	1
Diuretic	3	4	3	4
Arterial Vasodilator	2	2	2	2

Ace-I – Angiotensin Converting Enzyme Inhibitor, ARB – angiotensin receptor blocker,

DHCCB – dihydropyridine calcium channel blocker.

**Table 3:** Results of the Intervention Phases; Normally Distributed Data.

	3-Months Conventional HD	3-Months Daily HD	Paired-Difference		p-value
			Negative N(%)	Positive N(%)	
SBP pre-HD (mmHg; SD)	142(17)	139(14)	11(58)	8(42)	0.39
DBP pre-HD (mmHg; SD)	79(9)	80(9)	10(53)	9(47)	0.56
SBP post-HD (mmHg; SD)	127(19)	125(14)	8(42)	11(58)	0.40
DBP post-HD (mmHg; SD)	75(11)	76(8)	13(68)	6(32)	0.40
Dry Weight (kgs; SD)	77(14.4)	76.8(15.1)	9(47)	10(53)	0.70
Ultrafiltration/treatment (L; SD)	2.4(0.8)	1.3(0.6)	0(0)	19(100)	<0.001
Ultrafiltration rate (L/hr; SD)	0.60(0.20)	0.65(0.29)	12(63)	7(37)	0.17
ECFV (L; SD)	15.4(2.8)	15.2(3.2)	*11(65)	6(35)	0.77

SBP – systolic blood pressure, HD – hemodialysis, SD – standard deviation, DBP – diastolic blood pressure,

kgs – kilograms, L – liter, hr - hour;

*last value not carried forward for the 2 patients who withdrew.

**Table 4:** Results of the Intervention Phases; Non-Normally Distributed Data.

	‘N’	Paired Difference CHD-DHD Median (Quartiles)	Paired-Difference			p-value
			Negative N(%)	No change N(%)	Positive N(%)	
HTN Medication Intensity Score	19	1(0,2)	1(5.3)	7 (36.8)	11 (57.9)	0.01
Epinephrine, umol/L	10*	0(0,0)	1 (10)	8 (80)	1 (10)	1
Nor-Epinephrine, umol/L	15**	−0.2(−0.7, 0.3)	9 (60)	0	6 (40)	0.52
IL_6	12***	−0.35(−1.15, 0.29)	7 (58.3)	0	5 (41.7)	0.42
TBARS	12***	0.15(−0.65, 0.5)	5 (41.7)	0	7 (58.3)	0.83

* 7 samples had chromatographic interference - lab unable to do analysis, 2 patients withdrew from the study;

** 2 patients withdrew from the study, 2 samples were missed,

***started to measure these after the first 5 patients were already enrolled, 2 patients withdrew from the study.

Serious adverse events were rare during the trial. One patient was hospitalized during the study during the short daily hemodialysis phase with volume overload.

## Discussion

Short daily hemodialysis achieves a similar degree of systolic blood pressure control compared to conventional 3 times per week hemodialysis after a blood pressure optimization phase. However, this control is associated with a decreased requirement for antihypertensive medications. Our results highlight the importance of a run-in phase using a protocolized blood pressure algorithm when treatment modalities cannot be blinded.

The decline in blood pressure at the end of the 3 month run-in phase, compared to baseline, was likely multifactorial. Although we did not demonstrate a reduction in average post dialysis weight, there was tremendous variability with some participant's weight being decreased and other participant's weight being increased. Similar changes were seen in the anti-hypertensive medication use. A reduction in dialysate sodium has been associated with a reduction in blood pressure in hypertensive patients without a change in pre-dialysis serum sodium concentration in some [22], [23] but not all studies [24]. Post dialysis increases in serum sodium have been demonstrated for patients treated with conventional hemodialysis with dialysate sodium that is higher than the patient's serum sodium with a potential increase in thirst (occurs in 43% when dialysate sodium is 140 mmol/L, 20% when dialysate sodium is 138 mmol/L, [25]). It is unclear if the dietary instruction or the decrease in dialysate sodium in our study caused the small but statistically significant decrease in the total amount of ultrafiltration from the first month of the run in phase to the last month of the run in phase.

The mechanism for the decrease in antihypertensive requirements on short daily hemodialysis, compared to conventional hemodialysis, is unclear. A reduction in ECFV has been demonstrated in some [6], [9] but not all [7] studies as a mechanism contributing to the blood pressure lowering effect associated with short daily dialysis. Our study does not support this association for the population. There are a number of potential reasons for the differences in our results compared to other studies. To our knowledge a protocolized blood pressure management algorithm aimed at achieving an optimal dry weight prior to starting short daily hemodialysis has not been followed previously. Even during conventional hemodialysis ‘probing dry weight’ as was done in the DRIP trial was associated with approximately 7/3 mmHg decline in systolic and diastolic blood pressure respectively [26]. Also, given the inability to blind these modality studies, the possibility of differential patient management is possible.

The activity of the sympathetic nervous system was found to be important in a study that included 11 patients treated with short daily hemodialysis [6]. The investigators demonstrated a reduction in ultrafiltration volumes per session almost identical to those reported in our study. They hypothesized that large volume fluctuations could result in greater sympathetic nervous system stimulation. Greater amounts of ultrafiltration may also lead to increased post dialysis serum sodium which may stimulate the sympathetic nervous system [27]. We were unable demonstrate a difference in serum catecholamines with short daily compared to conventional hemodialysis but this does not rule out increase central sympathetic outflow.

In that last decade there has been an emerging evidence to support importance of oxidative stress in the development of hypertension [28], [29]. Hydrogen peroxide production (H_2_O_2_) was higher in granulocytes of patients with CKD treated with hemodialysis compared to controls [30]. The magnitude of ROS production by granulocytes and monocytes in that study population was significantly related to the plasma concentration of the lipid peroxidation product, malondialdehyde (MDA), which is commonly used as an indirect marker of oxidative stress [30]. H_2_O_2_ levels increased after hemodialysis with a cellulose acetate dialyzer highlighting the potential deleterious effects associated with the dialysis treatment itself [30]. We were unable to show a difference in serum T-BARs (measure of MDA) with short daily compared to conventional hemodialysis. Interestingly, phosphate has been shown to stimulate endothelial cell apoptosis which was characterized by increased oxidative stress [31]. It is unclear if the effects of an increase in dialysis frequency and exposure to dialysis tubing and membranes is counterbalanced by the improvements in serum phosphate that have been reported with short daily dialysis.

A generalized increase in inflammatory markers including IL-6 may occur via a number of mechanisms including volume overload and oxidative stress. We were unable to show a difference in IL-6 or albumin with short daily compared to conventional hemodialysis. In a prospective cohort study of 26 patients treated with short daily HD in-centre, Ayus et al reported significant improvements in hs-CRP values after 12 months [32]. In a cross-sectional study by Jefferies et al, lower CRP values were only seen for patients who were doing more frequent dialysis at home compared to those patients who were treated in-centre [33]. Post dialysis sodium, ultrafiltration volumes and ultrafiltration rates were much lower for the patients being treated at home compared to the patients being treated in-centre suggesting that post-dialysis increases in serum sodium and/or interdialytic ECFV expansion may explain these differences. They were unable to demonstrate lower IL-6 levels between patients undergoing conventional and short daily dialysis in-centre who had similar post dialysis serum sodium levels and ultrafiltration rates. The impact of more frequent dialysis on markers of oxidative stress and inflammation requires further study.

Our study has a number of limitations including the inability to blind the patients and investigators to the treatment group. We did not measure residual renal function and therefore could not assess the importance of this variable. However, the dialysis vintage of the patients suggests that many of them may have been anuric. Lastly, we did not measure ECFV at baseline and again at the end of the run-in phase; we are therefore unable to comment on the role changes in ECFV in the blood pressure improvement that was seen in the BP optimization phase. Strengths of our study over others that have been published to date include the run-in phase and use of standardized blood pressure algorithm.

In summary after a 3-month run-in phase in which blood pressure was optimized by decreasing dialysate sodium, adjusting dry weight and anti-hypertensive medications,

patients treated with short daily HD compared to conventional HD require fewer anti-hypertensive medications to achieve the same blood pressure. This effect on blood pressure control was not related to a reduction in ECFV, sympathetic nervous system activity, oxidative stress or inflammation. The mechanism(s) by which short daily HD allows for decreased use of anti-hypertensive medication remains unclear but may be related to effects on sodium balance and changes in peripheral vascular resistance that require further study.

## Supporting Information

Checklist S1 **CONSORT Checklist.**
(DOCX)

Protocol S1 **Trial Protocol.**
(DOC)
