# Supplementary material for: Short Daily versus Conventional Hemodialysis for Hypertensive Patients: A Randomized Cross-Over Study
Source: PLoS One. 2014 May 29;9(5):e97135. doi: 10.1371/journal.pone.0097135 (PMC4038634; doi:10.1371/journal.pone.0097135)
Supplement: Protocol S1 — Trial Protocol. (DOC) [file pone.0097135.s002.doc]

# What is the problem to be addressed?

# The problem of hypertension in patients with end stage renal disease

In the last Canadian Organ Replacement Register, the incident rate for end stage renal disease had increased from 102 per million population in 1993 to 158 per million population in 2002, an increase of 55% (1). While many options exist, the majority of patients with end stage renal disease will be treated with conventional (three times per week) in-centre hemodialysis that is estimated to cost approximately $60,000 per patient year (2,3). In addition to the tremendous cost to the health care system, the patients treated with hemodialysis have a reduced quality and quantity of life compared to the general population (4). More than 50% of the patients will die from cardiovascular disease; a risk 10-20 times greater than the general population (5).

Of the potentially modifiable cardiovascular risk factors, control of hypertension remains elusive. More than 80% of patients with end stage renal disease have hypertension; 70% of whom are poorly controlled using conventional therapy including salt restriction and medications (6). An expanded extracellular fluid volume and an increase in peripheral vascular resistance as a result of hemodynamic/trophic effects of an increased sympathetic nerve activity, angiotensin II, asymmetrical dimethyl arginine, and decreased nitric oxide are the most frequently quoted mechanisms contributing to hypertension in this population. The intermittent nature of conventional hemodialysis treatments (4 hours, 3 days/week) results in the majority of patients having a sustained expansion of the extracellular fluid volume that likely contributes to the activation of neurohormonal pathways. However, daily therapy including short daily hemodialysis (2 hours, 6 days/week) and nocturnal hemodialysis (6-8 hours, 5-6 days/week) improve or even normalize blood pressure. Short daily hemodialysis appears to improve blood pressure secondary to a reduction in extracellular fluid volume (7,8) whereas with the improvement in blood pressure with nocturnal hemodialysis occurs by a reduction in peripheral vascular resistance (8,9,10). This is consistent with the Katzarski et al experience (7-8 hours, 3 days/week) and one randomized controlled trial in which blood pressure control was due to normalization of extracellular fluid volume in some patients and a reduction in peripheral vascular resistance in others (11,12).

While daily hemodialysis has been explored as a mechanism for controlling hypertension predominately in North America, another approach in a few European centers has been to use *hemofiltration*. This method of blood purification is associated with an enhanced removal of toxic middle molecules compared to hemodialysis. Even when used only three times per week, hemofiltration has been associated with improvements in blood pressure (13) and cardiovascular stability (14-18).

In our one-month pilot study of daily hemofiltration, using a simplified delivery system, blood pressure was improved in spite of a 50% reduction in number of antihypertensive medications (19,20). However, it is unclear if the short-term improvement in blood pressure will be sustained. Patients may simply have been more compliant with their salt restriction leading to improved blood pressure given the short time frame of this study. In addition, a Hawthorne effect is possible. Lastly, mechanisms of improved blood pressure control were not assessed in our pilot study.

**1.2 Principle research questions to be addressed**

Primary objective: To determine if short daily hemofiltration, compared to conventional hemodialysis, is associated with a sustained (3month) improvement in systolic blood pressure.

Secondary objectives: 1) To determine if the mechanism by which short daily hemofiltration is associated with an improvement in blood pressure control is secondary to changes in sympathetic nervous system activity and/ or extracellular fluid volume 2) To determine if short daily hemofiltration, homeostasis (serum calcium, phosphate, potassium and bicarbonate), 3) To determine patient modality preference.

**1.3a Why is a clinical trial needed now?**

The results of the Hemodialysis Study (HEMO) suggest that further enhancement in the removal of the removal of small molecules such as urea is not associated with an improvement in outcomes for patients treated with conventional hemodialysis (21). Since cardiovascular disease is the most common cause of mortality in patients with ESRD, interventions that reduce patients’ risk for major adverse cardiovascular events have the greatest potential impact on patient morbidity and mortality (1). The results with short daily and nocturnal hemodialysis have been very encouraging yet both treatment modalities are complex and their widespread acceptance for use at home or for self-care has been very limited.

**1.3b HYPERTENSION AND CONVENTIONAL HEMODIALYSIS**

With the current standard of care, the majority of end stage renal disease patients treated with in-centre hemodialysis have toxic metabolites removed from their blood by diffusion 3 times per week, 3 to 4 hours per session. Uremic toxins and fluid are allowed to accumulate between sessions such that patients experience substantial fluctuations in urea and other solutes in addition to extracellular fluid volume. The treatment schedule is associated with hypertension in approximately 50-90% (22) of hemodialysis patients increasing the risk for left ventricular hypertrophy (23-25).

**Etiology of hypertension for patients on HD**

Hypertension in patients on hemodialysis is multifactorial. An expanded extracellular fluid volume is the most consistent finding in hypertensive hemodialysis patients. The high ultrafiltration rates required when patients dialyze only 3 to 4 hours 3 times per week often results in symptomatic hypotension during the treatment impeding the normalization of extracellular fluid volume. In unique studies by Charra et al., patients undergo dialysis 3 times per week for approximately 8 hours. Slow ultrafiltration (fluid removal) results in normalization of blood pressure in the majority of patients (26). However, other mechanisms such as increased sympathetic nervous system activity, angiotensin II, decreased nitric oxide etc likely contribute to the maintenance of hypertension in end stage renal disease, since ultrafiltration does not normalize BP in all patients (27,28). With regards to the sympathetic nervous system, studies utilizing plasma norepinephrine levels have been inconclusive in patients with end stage renal disease (29-34). This may be due to the fact that norepinephrine levels are a result of discharge, reuptake, metabolism and clearance. The limitations of plasma norepinephrine levels have been overcome with the use of microneurography, which is the only method of directly recording postganglionic muscle sympathetic nerve activity. Muscle sympathetic nerve activity has been found to be an important determinant of blood pressure in patients with chronic renal failure (35), on hemodialysis (36) and post renal transplant (37).

# Significance of hypertension for patients on HD

Regardless of the mechanism, hypertension has important implications for end stage renal disease patients. In a prospective cohort study of dialysis patients, Foley et al. showed that each 10mmHg rise in mean arterial pressure is independently associated with an increased risk of left ventricular hypertrophy (RR 1.48), the development of de novo cardiac failure (RR1.44) and de novo ischemic heart disease (RR 1.39) (38). In the studies by Charra et al., where blood pressure control is achieved by slow ultrafiltration, each 10mmHg increase in pre-dialysis mean arterial pressure was associated with a 39% increase in the risk of death (26). Similarly, each 10mmHg increase in post dialysis pulse pressure (systolic – diastolic pressure), has been associated with a 12% increase in the hazard for death (39). Additionally for patients on hemodialysis, pre-dialysis blood pressure was found to correlate with cerebral atrophy, suggesting that an improvement in blood pressure control may ultimately lead to a reduction in morbidity and mortality (40).

# Target blood pressure for patients on HD

In spite of the importance of hypertension for patients on hemodialysis, the most appropriate blood pressure target and duration of therapy that is required to reduce mortality is unknown. A U or J shaped mortality curve has been suggested in a number of different observational database driven studies (41,42). However, the correlation between low blood pressure and an increased risk of mortality appears to be an artifact of confounding due to reduced blood pressure caused by severe cardiovascular disease (42,43). Additionally, hypertension in the short term does not appear to increase the risk of death but re-emerges as an important risk factor with longer time on dialysis (44-47).

In summary, the concerns about a U or J shaped mortality curve are probably unjustified. Therefore based on the available information, the most recent Canadian Society of Nephrology guidelines have suggested a pre-dialysis blood pressure goal of <140/90 (48).

# Measurement of blood pressure for patients on hemodialysis

Accurate blood pressure measurements in hemodialysis units are difficult. Rahman et al compared ‘usual’ blood pressure measurements in 270 hemodialysis patients compared to those blood pressures measured using the standardized American Heart Association protocol. The pre-dialysis usual blood pressure measurement over estimated systolic/diastolic blood pressure by 14.3/7mmHg emphasizing the need for standardized measurements (49). Similar results have been seen with ambulatory blood pressure measurements. However, an average of 2 weeks of pre-dialysis blood pressure of >150/85 has a sensitivity of 80% in the diagnosis of hypertension compared to ambulatory blood pressure measurements (50). Additionally, an average of 4 weeks of pre-dialysis blood pressures when compared to ambulatory blood pressure monitoring is equally effective in explaining the variance in LV mass (51,52). However, the average blood pressure measurement does not allow for the assessment of non-dipping of nocturnal blood pressure that has been associated with an increase in LV diameter and mortality in ESRD patients (53-56).

In summary, although ambulatory blood pressure monitoring allows for a more complete assessment of hypertension, an average of 2-4 weeks of pre-dialysis blood pressure appears to be an effective alterative for routine monitoring of blood pressure. Additionally, the use of standardized techniques for blood pressure measurement are critical to avoid mislabeling of patients.

# Pharmacological methods for blood pressure control on hemodialysis

With the exception of carvedilol in congestive heart failure, there are no published randomized prospective controlled trials of the benefits of specific classes of anti-hypertensive medications, and retrospective studies have yielded conflicting results in patients with end stage renal disease (57-60).

However, several classes of antihypertensive medications can affect muscle sympathetic nerve activity results including sympatholytics, angiotensin converting enzyme inhibitors and angiotensin receptor blockers (61).

**1.3d DAILY HEMODIALYSIS**

Daily hemodialysis schedules can be either short daily (2 hours 6 days per week) or nocturnal (6-9 hours, 5-6 days per week). By increasing dialysis frequency but maintaining overall dialysis weekly time, daily Kt/V (K=urea clearance in mls/min, t=treatment time in minutes, volume of patients in mls) is reduced but standard weekly Kt/V is increased (62). This change in frequency of treatment has been associated with improvements in blood pressure control (7, 63-66) and left ventricular hypertrophy (7,68) when compared to conventional hemodialysis. In addition, most patients will experience an improvement in quality of life due to a reduction in dialysis related symptoms (65,66). Similarly, nocturnal hemodialysis has been associated with improvements in blood pressure (8), left ventricular hypertrophy (9) and quality of life (3,67).

**1.3e MECHANISMS OF BLOOD PRESSURE CONTROL WITH DAILY HEMODIALYSIS**

Short daily and nocturnal hemodialysis appear to have different mechanisms of blood pressure control. In a randomized crossover study of 12 patients comparing conventional hemodialysis to short daily hemodialysis, extracellular water as measured by bioimpedance, decreased from 52.7(±11.4)% to 47.6(±7.5)%. Extracellular water content positively correlated with 24-hour systolic blood pressure and left ventricular mass index (7). Similarly, patients treated with short daily hemodialysis in the London Daily/Nocturnal Study also had a decrease in extracellular fluid volume measured by bioimpedance spectroscopy (8). However, the patients treated with nocturnal hemodialysis did not have a decrease in extracellular fluid volume and the authors speculated that a different antihypertensive mechanism must be responsible for the decline in blood pressure. Their results were supported by another study in which 28 patients were converted from conventional hemodialysis to nocturnal hemodialysis and experienced a significant improvement in their blood pressure but no change in the extracellular fluid volume (9). Subsequently, after conversion from conventional to nocturnal hemodialysis, 18 patients experienced a decrease in peripheral vascular resistance, which was attributed to a decrease in sympathetic nervous system activity (10).

**1.3f LIMITATIONS TO DAILY HEMODIALYSIS**

However, there are limitations to daily hemodialysis. In centre therapy is not sustainable for daily nocturnal hemodialysis as patients would be required to sleep at the hospital. The increased nursing preparation time required for setting up/disconnecting 2 patients instead of 1 in a 4 hour time slot for short daily hemodialysis is also potentially problematic (68). In addition, overall costs including centre costs, nursing, medications, and hospitalizations are higher for in-centre hemodialysis than hemodialysis performed at home (3,68). The obvious solution to address the nursing shortage and cost issues is to have capable end stage renal disease patients perform their own treatment at home. This approach is also problematic. Most hemodialysis machines are complex and require 6-8 weeks of patient training time before they are able to perform their own treatment. The treatment is further complicated by the need to utilize large quantities of water that is of suitable quality for dialysis. Patients must learn how to run and maintain reverse osmosis machines with the attendant increase in cost, complexity and time. The patient’s home also requires modifications to existing electrical and plumbing systems. Additional water and electrical costs are either borne by the patient or their end stage renal disease program. The time commitment required by the patient to undertake their own treatment is extensive due to the set up (45-60mins), treatment and take down/disinfection times (45-60mins) such that the entire short daily treatment takes approximately 4 hours. In both Canada and the United States, the additional cost of materials (despite being offset by the decrease in nursing and hospitalization costs) is not remunerated to the end stage renal disease programs. Thus, due to these limitations, less than 1% of patients are treated with home hemodialysis in the United States (69).

**1.3g HEMOFILTRATION**

Hemofiltration uses convection for the removal of molecules. This different mechanism of solute removal tends to be associated with reduced clearance of small molecules but enhanced clearance of middle molecules compared to hemodialysis. **The technology required for hemofiltration is more complicated and more expensive than hemodialysis.** Perhaps for this reason, the use of hemofiltration has remained limited to a few centers in Europe that are capable of producing sterile solution for infusion on-line. Those centers that used **thrice-weekly** hemofiltration gradually increased the amount of replacement solution from 18-20L/treatment (Kt/V ~0.45) to 40L or more (Kt/V~1.0) (70). Despite the initial ‘low volumes’ of replacement solution, a number of investigators were able to demonstrate a reduction in hypertension and improved cardiovascular stability (less hypotension during and after treatment). In high- risk patients (diabetes mellitus and elderly), there was a reduction in morbidity (hospitalizations for hypotension/hypertension/myocardial insufficiency; 13.5 versus 9.2 days/year) and mortality for patients treated with hemofiltration compared to hemodialysis (71-75). For those patients who had experienced both hemodialysis and hemofiltration, there was an overwhelming preference for hemofiltration predominately due to patient stability during the treatment (76). Despite the very encouraging early results with hemofiltration, hemodialysis remains the predominant modality in North America likely due to the complexity of producing large volumes of sterile infusion solution that is required for conventional three times per week hemofiltration.

**1.3h DAILY HEMOFILTRATION – Our preliminary pilot data**

Daily hemofiltration has the potential to combine the benefits of both short daily hemodialysis and conventional hemofiltration. However, experience with daily hemofiltration outside of the intensive care unit is limited. We undertook a pilot project using a new system developed by NxStage Medical (Lawrence, MA). The system consists of a simple portable microprocessor controlled cycler for therapy administration and a disposable cartridge for blood and fluid handling (Appendix 1,2). The system is used with pre-packaged solutions much like peritoneal dialysis such that additional plumbing and water requirements are not necessary in the home. Because of the simplicity of the system, patient training time is reduced to approximately 3 weeks.

For the purposes of our prospective pilot study, urea clearance was targeted at 0.40+/-0.5 per treatment 6 days a week in keeping with a standard Kt/V of 2.0. In spite of the lower weekly urea clearance delivered, the patients experienced a reduction blood pressure (Table 1), a 50% reduction in the number of antihypertensive medications, an improvement in quality of life, and in beta-2-microglobulin levels, a marker of middle molecular weight clearance (19,20). However, the study duration was too short to determine if the improvement in blood pressure will be maintained in the long term. In addition, we did not explore the mechanism(s) by which the blood pressure was improved.

Table 1: Improvements in Blood Pressure with Daily Hemofiltration (6x/week) Compared to Conventional Hemodialysis N=7

| Pre Treatment Pressure | Hemodialysis | Hemofiltration | p-value |
| --- | --- | --- | --- |
| Mean Arterial Pressure (mmHg) | 96 (±11) | 86 (±12) | 0.001 |
| Systolic Blood Pressure  (mmHg) | 139 (±18) | 128 (±19) | 0.001 |
| Diastolic Blood Pressure  (mmHg) | 74 (± 9) | 66 (±11) | 0.01 |

**1.3k SUMMARY AND RATIONALE**

The number of patients with end stage renal disease continues to increase. Recent studies would suggest that conventional hemodialysis is not likely the best therapeutic dialysis option for our patients. The risk of death from cardiovascular disease is 10-20 times greater for patients with end stage renal disease than in the general population. Of the potentially modifiable cardiovascular risk factors, hypertension is present in the majority of patients and is poorly controlled using standard antihypertensive therapy. Daily hemodialysis therapies have been very successful in reducing blood pressure but the complexity of the treatment limits access to a minority of patients. We believe that daily hemofiltration represents an improved treatment modality for patients with end stage renal disease. Therefore, we propose a randomized cross-over study to compare the effect of daily hemofiltration, using a simplified method of delivery, to conventional hemodialysis on blood pressure.

**1.4 References to systematic reviews; discuss need for a trial in this light**

Previous reviews and one systematic review have been written on the benefits of daily dialysis (78-80). The two reviews on daily hemofiltarion was based on our pilot study (81,82). One review has compared conventional hemodiafiltration with conventional hemodialysis but concluded that there wasn’t enough data to determine the optimal treatment modality (83).

**1.5 How will the results of the trial be used?**

The results of this study may support the use of daily hemofiltration as an acceptable alternative to conventional hemodialysis. If the primary hypothesis is supported, the results of this study will be used to support the application of a large multi-centre controlled trial examining the effects of daily hemofiltration compared to conventional hemodialysis on the ‘hard’ major adverse cardiovascular end-points (stroke, non-fatal myocardial infarction and cardiovascular death) and soft major adverse cardiovascular end-points (acute coronary syndrome, congestive heart failure, angina, revascularization, transient ischemic accident).

**2.1 Proposed trial design –**Randomized cross-over trial of end stage renal disease patients with resistant hypertension

**2.2 Planned trial interventions**

The study will be a 9-month randomized, unblinded, crossover trial of patients comparing short daily (6 days/week) in-centre hemofiltration with a spKt/V (measure of dialysis adequacy: sp=single pool, K= dialyzer clearance of urea, t= time on dialysis, V=volume of urea distribution) of 0.5 (±0.05) to conventional hemodialysis with a spKt/V of 1.2-1.4 yielding approximately equivalent standard Kt/V measurements. A pre and post treatment blood pressure will be measured each treatment, using standard techniques (*see below*). The number, type and strength of antihypertensive medications will be recorded at study entry and at the beginning of each month. Standard laboratory parameters for metabolic control (hemoglobin, calcium, phosphate, albumin, electrolytes, creatinine and urea) will be collected monthly. Additionally, each patient will have an assessment of extracellular fluid volume via bioimpedance, sympathetic nervous system activity via muscle sympathetic nerve activity and ambulatory blood pressure monitoring on 3 separate occasions.

**2.3 Proposed practical arrangements for allocating participants to the trial**

All eligible hemodialysis patients within the Ottawa Hospital will be provided with an information letter about the proposed study. Patients who are interested in participating in the study will be invited to attend an information session. Those patients who meet study criteria and sign informed consent will start training for self-care daily (6 days/week) hemofiltration on a first come first serve basis after completing a further 3 months of hemodialysis.

**2.4 Methods for protecting against sources of bias**

Patients cannot be blinded to the hemofiltration modality. However, the final data analysis will be by individuals who are blinded to the patient’s therapy. Nurses who are unaware of the objectives of the study will take vital signs with an automated blood pressure cuff. Ambulatory blood pressure monitoring and muscle sympathetic nerve activity recordings are unlikely to be affected by patient knowledge of treatment modality.

**2.5 Planned inclusion/exclusion criteria**

Inclusion criteria: All prevalent hemodialysis patients (on therapy for greater than 3 months) will be eligible for the study if: 1) They have systolic hypertension with a blood pressure >140mmHg pre-dialysis and are on 3 or more antihypertensive medications 2) They have a well functioning vascular access capable of maintaining blood flows of 350 mls/minute 3) They are able to make the time commitment for daily therapy 4) They are capable of giving informed consent.

Exclusion criteria: Patients will be excluded if: 1) They are expected to receive a transplant within the next 12 months 2) If they are considering a switch to peritoneal dialysis 3) They are not expected to survive 12 months 4) They have had a serum potassium value of 6.6 mEq/L or greater in the 90 days preceding enrollment, based on 3 separate monthly measurements 5) They have infections that require isolation (Vancomycin Resistant Enterococcus, Methicillin Resistant Staphylococcus Aureus, Hepatitis B) 6) They have known symptomatic dilated cardiomyopathy (New York Association Class II or III with left ventricle ejection fraction of <0.35

**2.6 Proposed duration of treatment –** 9 months

**2.7 Proposed frequency and duration of follow-up –** The total duration of treatment is 9 months. Patients will be seen at least once per week by their attending nephrologist as is the standard of care in the Ottawa Hospital. The research coordinator will be responsible for adjusting the prescription in hemodialysis and hemofiltration to achieve the Kt/V targets.

**2.8 Proposed primary and secondary outcome measures**

Primary objective: To determine if short daily hemofiltration, compared to conventional hemodialysis, is associated with a sustained (> 3months) improvement in systolic blood pressure.

Secondary objectives: 1) To determine if the mechanism by which short daily hemofiltration is associated with an improvement in blood pressure control is secondary to changes in sympathetic nervous system and/ or extracellular fluid volume 2) To determine if short daily hemofiltration, compared to conventional hemodialysis maintains metabolic homeostasis (serum calcium, phosphate, potassium and bicarbonate), and 3) To determine patient modality preference.

**2.9 Measurement of outcomes at follow-up (Appendix 3)**

After providing informed consent, baseline medication use will be recorded and the comorbidity score (Charleson) calculated. Laboratory values including the standard hemoglobin, calcium, phosphate, albumin, electrolytes, creatinine, and urea, will be measured at study entry. Each patient will continue hemodialysis for an additional 3 months. During that time, the patient’s Kt/V will be adjusted to 1.2-1.4. If the Kt/V is too high, the prescribed blood pump speed will be reduced. If necessary, the patient’s current dialyzer will be exchanged for a different polysulfone dialyzer with a lower urea clearance. Lastly, the patient’s time may be decreased by a maximum of 30 minutes as long as this does not interfere with fluid removal (mimic the HEMO study). During this time because of the effects on muscle sympathetic nervous system activity, any patients who are taking sympatholytics, angiotensin converting enzyme inhibitors or angiotensin receptor blockers will have these medications weaned. If the patient’s blood pressure increases to greater than 165/95, metoprolol, long acting dihydropyriding calcium channel blocker and / or non-dihydropyridine calcium channel blocker (with known minimal effect on central sympathetic outflow) will be introduced (Appendix 4). All patients will have their dry weight adjusted in an attempt to decrease blood pressure pre-dialysis to <140/90). Standard laboratory tests will be repeated monthly (first Wednesday or Thursday) and medications adjusted to meet currently accepted targets for hemoglobin, calcium and phosphate (Appendix 5). The prior 3 months of hemodialysis therapy, within a clinical trial setting, will ensure optimal management of blood pressure prior to starting hemofiltration to ensure that any improvements in BP on daily HF are secondary to modality and not more intensive intervention from health care providers*.*

After the completion of the 3 month hemodialysis run in phase, patients will be randomized to either to a further 3 months of hemodialysis or 3 months of hemofiltration. The starting prescription for hemofiltration will be enough replacement fluid (Hemosol) to achieve a daily (6 days/week) treatment Kt/V of 0.50 (±0.05). Each treatment will take approximately 2-2.5 hours and will occur during the day in centre. Remaining in-centre for the duration of the study avoids the potential bias of typically lower blood pressures at home compared to the clinic environment. The starting prescription for hemodialysis will be unchanged from the preceding 3 months (3 days/week, approximately 3.5-4 hours per treatment). Each patient will undergo measurement of their extracellular fluid volume using bioelectrical impedance, sympathetic nervous system activity using microneurography and ambulatory 24-hour blood pressure measurements (during an off treatment day) prior to the start of and at the end of this 3 month phase. Additionally, each patient will have the standard laboratory investigations repeated on the first Wednesday/Thursday of the month. The nurses will document vitals pre and post each treatment session. Medications, and any adverse events including hospitalizations will be reassessed at the time of monthly blood work.

After completing the 3 months of hemofiltration or hemodialysis, patients will then switch treatment modality for a further 3 months. The same protocol will be followed for laboratory investigations, assessment of vitals, medications and adverse events as during the preceding 3 months. Repeat extracellular fluid volume measurement, muscle sympathetic nerve activity and ambulatory blood pressure recordings will be completed at the end of the study.

**2.9a BLOOD PRESSURE MEASUREMENTS**

Blood pressure will be measured pre and post dialysis as recommended by the Canadian Hypertension Society (www.hypertension.ca). Briefly, the patient will have their back supported, feet on the floor and will sit quietly for 5 minutes. The arm will be supported and a mercury sphygmomanometer will be used*.* An appropriate sized cuff will be placed at the level of the heart and inflated to 20mmHg above the level at which the radial pulse disappears. Phase I Korotkoff, appearance of first sound, will be taken as the systolic blood pressure and phase V Korotkoff, disappearance of sound, will be taken as the diastolic blood pressure. Two measurements will be taken and averaged. Patients will be discouraged from using alcohol, caffeine, or tobacco within one hour of BP measurement. The measurements will be taken in the arm opposite to the one with a functioning access (if a central venous catheter is in place, the non-dominant arm will be used) prior to needle insertion for the hemodialysis/hemofiltration treatment.

Ambulatory blood pressures will be performed with a portable oscillometric recorder (Spacelabs 90207). Recordings will begin after the completion of hemodialysis and hemofiltration and will continue for a 44 hour period (the time between treatments) on three different occasions (at the time of randomization, after the first 3 months of the cross-over phase and again at study completion). The pressure will be measured every 30 minutes. The most appropriate cuff will be selected from the 4 sizes supplied by the manufacturer Sleep times will be determined from patient’s diaries. Nocturnal hypertension will be defined as the absence of a decrease of blood pressure during sleep by 10% compared to the awake period. If fewer than 75% of the potential readings are recorded or if more than 90 minutes lapses without a valid blood pressure recording, the ambulatory session will be repeated in 7 days.

**2.9b LABORATORY MEASUREMENTS**

The standard laboratory tests including hemoglobin, electrolytes, bicarbonate (TCO2), calcium, albumin, phosphate, urea and creatinine (Jaffe) will be measured pre hemodialysis/hemofiltration using the Ottawa Hospital Laboratory.

**2.9c EXTRACELLULAR FLUID VOLUME MEASUREMENT BY BIOIMPEDANCE**

For patients on hemodialysis, an increase in extracellular fluid volume is associated with hypertension and left ventricular hypertrophy (84). Tracer dilution techniques are considered the gold standard for the measurement of extracellular water but are complicated and not available in many centres. Additionally, extracellular water assessment by the bromide technique in patients with an expanded extracellular water is problematic due to the uncertainty of the correction constants for plasma water and the Gibbs-Donnan equilibrium (85). Additionally, a number of different clinical assessment tools have been used to estimate a patient’s hydration status including physical examination, blood volume monitoring and inferior vena cava measurement, but they either do no correlate well with direct measures of extracellular fluid volume and/or cannot be used to track sequential changes in volume status (86-90). However, bioelectrical impedance technology offers the potential to directly assess extracellular fluid volume, intracellular fluid volume and total body water. The technology is based on the ability to detect differences in the conductive properties of a cell by measuring its resistance (impedance) to electrical current. At low frequencies, the current cannot cross cell membranes and hence is only flowing through the extracellular fluid volume. At high frequencies, the current flows through the extracellular fluid volume and intracellular fluid volume. The technique is reliable for tracking sequential changes in extracellular fluid volume (r=0.95 between measurements on two separate days, 85).

For the purposes of this study, post treatment extracellular fluid volume will be measured 3 times; at the time of randomization, after the first 3 months of the cross-over phase and again at study completion using the Xitron Hydra (Xitron Technologies, San Diego, CA). The Hydra is a multifrequency bioimpedance analyzer that allows for differentiation of extracellular fluid volume and intracellular fluid volume. Four electrodes will be placed on the wrist and ankle on the non-fistula side. The measurement will take place 20 minutes after completion of the hemodialysis or hemofiltration treatment to allow for plasma re-filling. Previously the coefficient of variation of the method and intra-observer error were 2.5% (91). One of the investigators (DZ) will obtain expertise in the technique with the assistance of Dr. C. Chan at the University Health Network, Toronto, Ontario.

**2.9d SYMPATHETIC NERVE ACTIVITY**

As previously described by the co-applicant (MR), postganglionic multifibre muscle sympathetic nerve activity will be recorded microneurographically using a sterile tungsten microelectrode with a shaft diameter of 200 μm and a non-insulated tip of 5 μm in diameter inserted into a muscle fascicle of the peroneal nerve posterior to the fibular head. A reference electrode is inserted subcutaneously 1 – 2 cm from the recording electrode. Electrodes will be connected to the remote, low noise preamplifier of the Nerve Traffic Analyzer, (University of Iowa, Iowa City, Iowa). The neural signal will be amplified by 40,000, input to a bandpass filter (0.5 to 1 KHz) and integrated to obtain a mean voltage neurogram. The mean voltage neurogram, ECG, respirations and BP signals will be digitized with a sampling frequency of 2,000 to 5,000 Hz, and recorded on a Ponemah Data Acquisition System (Gould, Valley View, OH). Pulse synchronicity and low signal-to-noise ratio will confirm burst activity as being of muscle sympathetic efferent origin. MSNA (burst/min and bursts/100 beats) will be calculated from 10-minute recordings after 20 minutes of rest. Muscle sympathetic nerve activity recordings will be performed at the time of randomization, after the first 3 months of the cross-over phase and again at study completion when the patient is at their dry weight.

**2.10 What is the proposed sample size?** 20 patients. Based on our previous pilot study, assuming a standard deviation of 11, an alpha = 0.05, we will have 80% power to detect a difference of 11 between the groups.

**2.11 Planned recruitment rate –** Patients will be recruited from the ~ 600 patients undergoing in-centre hemodialysis at the Ottawa Hospital. Due to limited access to the machines for hemofiltration a maximum of 10 patients can be treated in each 6 month period. Allowing for upfront recruitment and training time, it is anticipated that it will take 18 months to recruit all of the patients with a further six months after the last patient is recruited to complete the study. Data analysis and manuscript preparation are expected to take an additional 6 months.

**2.12** **Anticipated compliance and loss to follow-up problems –** Reported drop out rates from daily dialysis programs are low. In our own home hemodialysis program, only 1 patient out of 20 has chosen to return to conventional hemodialysis after completing training. In our one-month pilot study of daily hemofiltration, one patient dropped out of the study due to an adverse event. Given the longer duration of follow-up and the requirement for an in-centre protocol, we anticipate 2 patients may drop out of the study. Furthermore, we don’t anticipate any loss to follow-up as end stage renal disease patients require dialysis as a life saving procedure. However, patients may decide to withdraw from the study.

**2.13 Proposed type of analysis**

The primary analysis, using a t-test, will be a comparison of mean pre-dialysis systolic blood pressure values from the last month on hemodialysis and the last month on hemofiltration. As described by Zoccali et al, this will give us an assessment of the pressure load as experienced by the left ventricle (92). Supporting analytic and descriptive analysis will be carried out to ensure Consort guidelines can be followed for publication of results.

Continuous outcomes measures for the secondary analysis will be analyzed in exactly the same manner. All efforts will be made to minimize missing values. A sensitivity analysis under extreme but reasonable assumptions will be carried out to evaluate the impact of any missing values.

Lastly, linear regression will be undertaken with systolic pressure as the dependent variable. Treatment type, extracellular fluid volume and sympathetic nervous system activity will be examined for their independent effects on blood pressure control.

**2.14 Feasibility**

In a review of our hemodialysis program, 85 patients were identified who fulfilled criteria for resistant hypertension (3 or more drugs on 3 consecutive runs, blood pressure >140/90). Assuming that 50% of patients would be potentially interested in participating in the study, we will have enough study candidates. However, we are concerned that patients may not want to return to hemodialysis after daily hemofiltration and have therefore limited the study duration to 9 months. Patients will also have the opportunity to select the modality of their choice as their long-term treatment option upon completion of the study. Lastly, we have Health Canada approval to use Normocarb for this study but have sent in an amendment to be able to use Hemosol as this solution is available in larger bags and comes premixed decreasing the nursing workload.

**3.1-3.3 Trial Management**

Principal applicant: I am an Assistant Professor in the Division of Nephrology, Department of Medicine at the Ottawa Hospital and the Kidney Research Centre, Ottawa Health Research Institute, University of Ottawa. I have a MSc in Clinical Epidemiology and have been a principle and site investigator for a number of clinical studies. My interest has been in daily therapies as the Director of the Home Hemodialysis Program and in cardiovascular morbidity and mortality. I am responsible for the design and implementation of the protocol. This involves supervision of the research team to prevent protocol violations and to ensure strict adherence to ICH Good Clinical Practice Guidelines. I have the necessary protected time and full support of the Kidney Research Centre Staff.

Kidney Research Centre Staff: These individuals have extensive clinical trials experience with a research manager, several research coordinators and clerks for data entry. The research coordinator will be the same individual that assisted with the pilot hemofiltration study.

Co- Investigators: Dr. Kevin Burns is a nephrologist with the Ottawa Hospital, Research Director for the Kidney Research Centre and a Professor with Department of Medicine, University of Ottawa. He is a Senior Scientist at the OHRI. He participated in our first study of daily hemofiltration and has been invited to speak on the topic. He will be involved in all aspects of trial management. Dr. Marcel Ruzicka is a nephrologist with the Ottawa Hospital and is the director of the Renal Hypertension Program. He has published experience in peroneal nerve microneurography used for the assessment of MSNA. He will be involved in all aspects of trial management. Dr. Paul Hebert is an intensivist within the Ottawa Hospital, senior scientist with the OHRI and has extensive clinical trials experience. He has assisted with the trial design and will provide ongoing support throughout the study. Dr. Dean Fergusson is a methodologist and scientist with the OHRI and has also assisted in trial design and will provide ongoing support throughout the study.

**REFERENCES**

1. 2002 Report, Volume 1: Dialysis and Renal Transplantation, Canadian Organ Replacement Register, Canadian Institute for Health Information, Ottawa, Ontario, 2002
2. McFarlane PA, Pierratos A, Redelmeier. Cost savings of home nocturnal versus conventional in-center hemodialysis. Kidney Int 2002; 62: 2216-2222.
3. Mohr PE, Neumann PJ, Franco SJ, Marainen J, Lockridge R, Ting G. The case for daily dialysis: its impact on costs and quality of life. Am J Kidney Dis 2001; 37(4): 777-789.
4. Laupacis A, Keown P, Pus N, Drueger H, Ferguson B, Wong C, Muirhead N. A study of the quality of life and const-utility of renal transplant. Kidney Int 1996; 50(1): 235-42.
5. Foley RN, Parfrey PS, Harnett JD, Kent GM, Murray DC, Barre PE. The prognositc importance of left ventricular geometry in uremic cardiomyopathy. J Am Soc Nephrol 1995; 5(12): 2024-31
6. Agarwal r, Nissenson AR, Batlle D, Coyne DW, Trout JR, Warnock DG. Prevalence, treatment, and control of hypertension in chronic hemodialysis patients in the United States. Am J Med 2003; 115; 291-297
7. Fagugli RM, Reboldi G, Quintaliani g, Pasini P, Ciao g, Cicconi B, Pasticci F, Kaufman JM, Buoncristiani U. Short daily hemodialysis: blood pressure control and left ventricular mass reduction in hypertensive hemodialysis patients. Am J Kidney Dis 2001, 38(2): 371-376.
8. Nesrallah G, Suri R, Moist L, Kortas C, Lindsay RM. Volume control and blood pressure management in patients undergoing quotidian hemodialysis. Am J Kidney Dis 2003; 42(1): S13-17
9. Chan CT, Floras JS, Miller JA, Richardson MA, Pierratos A. Regression of left ventricular hypertrophy after conversion to nocturnal hemodialysis. Kidney Int 2002; 61: 2235-2239.
10. Chan CT, Harvey PJ, Picton P, Pierratos A, Miller JA, Floras JS. Short-term blood pressure, noradrenergic, and vascular effects of nocturnal home hemodialysis. Hypertension 2003; 42: 925-31
11. Katzarski KS, Charra B, Luik AJ, Nisell J, Filho JCD, Leypoldt JK, Leunissen KML. Laurent G, Bergstrom J. Fluid state and blood pressure control in patients treated with long and short haemodialysis. Nephrol Dial Transplant 1999; 14: 369-375
12. Luik AJ, Dande FM, Weideman P, Cheriex E, Looman JP, Leunissen KML. The influence of increasing the dialysis treatment time and reducing dry weight on blood pressure control in hemodialysis patients: A prospective study. Am J Nephrol 2001; 21: 471-478
13. Quellhorst E, Schuenemann B, Borghardt J: Clinical and technical aspects of hemofiltration. Artif Organs 1978; 2(4): 334-338
14. Collins AJ, Keshaviah P, Ilstrup KM, Shapiro F: Clinical comparison of hemodialysis and hemofiltration. Kidney Int 1985; 28(suppl 17): S18-S22.
15. Altieri P, Sorba GB, Bolasco PG, Bostrom M, Asproni E, Ferrara R, Bolasco F,Cassu M, Cadinu F, Cabiddu GF, Casu D, Ganadu M, Passaghe M, Pinna M: On-line predilution hemofiltration versus ultrapure high-flux hemodialysis: a multicenter prospective study in 23 patients. Sardinian Collaborative Study Group of On-Line Hemofiltration. Blood Purif 1997; 15(3): 169-181
16. Altieri P, Sorba G, Bolasco P, Asproni E, Ledebo I, Cossu M, Ferrara R, Ganadu M, Cadinu F, Serra G, Cabiddu G, Sau G, Casu D, Passaghe M, Balasco F, Pitis R, Ghisu T: Predilution haemofiltration – the second sardinial multicentre study: comparisons between haemofiltation and haemodialysis during identical kt/v and session times in a long-term cross-over study. Nephrol Dial Transplant 2001; 16: 1207-1213.
17. Pierides AM, Schniepp B, Johnson WB. Hemofiltration in the treatment of acute and chronic renal failure. Proc Dial Transplant Forum 1979; 50-53.
18. Schaefer K, v. Herrath D, Gullberg CA, Asmus G, Hufler M, Offermann G, Cremer H, Heuck CC, Ritz E. Chronic hemofiltration a critical evaluation of a new method for the treatment of blood. Artif Organs 1978; 2(4): 386-394.
19. Zimmerman DL, Swedko PJ, Posen GA, Burns KD. Daily hemofiltration with a simplified method of delivery. ASAIO J 2003; 49(4): 426-429
20. Jaber BL, Zimmerman DL, Teehan GS, Swedko P, Burns K, Meyer KB, Leypoldt JK. Daily Hemofiltration for End-Stage Renal Disease: A Feasibility and Efficacy Trial. Blood Purif 2004; 22: 481-489.
21. Eknoyan G, Beck GJ, Cheung AK, Daugirdas JT, Greene T, Kusek JW, Allon M, Bailey J, Delmez JA, Depner TA, Dwyer JT, Levey AS, Levin NW, Milford E, Ornt DB, Rocco MV, Schulman G, Schwab SJ, Teehan BP, Toto R: Effect of dialysis dose and membrane flux in maintenance hemodialysis. N Engl J Med 2002; 347(25): 2010-2019
22. Horl MP, Horl WH. Hemodialysis-associated hypertension: pathophysiology and therapy. Am J Kidney Dis 39(2) 2002:227-244
23. Stack AG, Saran R. Clinical correlates and mortality impact of left ventricular hypertrophy among new ESRD patients in the United States. Am J Kidney Dis 2002; 40(6): 1202-10
24. London GM, Pannier B, Guerin AP, Blacher J, Marchais SJ, Darne B, Metivier F, Adda H, Safar ME. Alterations of left ventricular hypertrophy in survival of patients receiving hemodialysis: follow-up of an interventional study. J Am Soc Nephrol 2001; 12(12): 2759-67
25. Foley RN, Parfrey PS, Sarnak MJ. Epidemiology of cardiovascular disease in chronic renal disease. J Am Soc Nephrol 1988; (12 suppl): S16-23
26. Charra B, Calemard E, Laurent G. Importance of treatment time and blood pressure control in achieving long-term survival on dialysis. Am J Nephrol 1996; 16: 35-44
27. Salem M. Hypertension in hemodialysis populations? High time for answers. Am J Kidney Dis 1999; 33: 592-594).
28. Converse RL, Jacobsen TN, Toto RD, Jost CM, Cosentino F, Fouad-Tarazi F, Victor RG. Sympathetic overactivity in patients with chronic renal failure. N Engl J Med 1992; 327 (27): 1912-1918
29. Nies AS, Robertson D, Stone WJ. Hemodialysis Hypotension is not the result of uremic peripheral autonomic neuropathy. J Lab Clin Med 1979; 94: 395-402
30. Textor SC, Gavras H, Tifft CP, Bernard DB, Idelson B, Brunner HR. Norepinephrine and renin activity in chronic renal failure. Evidence for interacting roles in hemodialysis hypertension. Hypertension 1981; 3: 294-299
31. Corder CN, Sharma J, McDonald RH. Variable levels of plasma catecholamines and dopamine beta-hydroxylase in hemodialysis patients. Nephron 1980; 25: 267-272
32. Cuche JL, Prinseau J, Selz F, Ruget G, Gablin A. Plasm free, sulfo- and glucuro-conjugated catecholamines in uremic patients. Kidney Int 1986; 30: 566-572
33. Campese VM, Romoff MS, Levitan D, LaneK, Massry SG. Mechanisms of autonomic nervous system dysfunction in uremia. Kidney Int 1981; 20: 246-253
34. Henrich WL, Katz FH, Molinoff PB, Schrier RW. Competitive effects of hypokalemia and volume depletion on plasma renin activity, aldosterone and catecholamine concentrations in hemodialysis patients. Kidney Int 1977; 12: 279-284
35. Ligtenberg G, Blankestijn PJ, Oey PL,Klein IHH, Dijkhorst-Oei LT, Boosma F, Wieneke GH, vanHuffelen AL, Loomans HA. Reduction of sympathetic hyperactivity by enalapril in patients with chronic renal failure. N Engl J Med 1999; 340: 1321-1328

5

1. Converse RL, Jacobsen TN, Toto RD, Jost CM, Cosentino F, Fouad-Tarazi F, Victor RG. Sympathetic overactivity in patients with chronic renal failure. N Engl J Med 1992; 327(27): 1912-1918
2. Hausberg M, Kosch M, Harmelink P, Barenbrock M, Hohage H, Kesters K, Dietl KH, Rhan KH: Sympathetic nerve activity in end-stage renal disease. Circulation 2002; 106(15): 1974-1979
3. Foley RN, Parfrey PS, Harnett JD, Kent GM, Murray DC, Barre PE: Impact of hypertension on cardiomyopathy, morbidity and mortality in end-stage renal disease. Kidney Int 1996; 49: 1379-1385
4. Klassen PS, Lowrie EG, Reddan DN, DeLong ER, Coladonato JA, Szczech LA, Lazarus JM, Owen WR. Association between pulse pressure and mortality in patients undergoing maintenance hemodialysis. JAMA 2002; 287(12): 1548-1555
5. Savazzi GM, Cusmano F, Bergamaschi E, Vinci S, Allergri L, Garini G. Hypertension as an etiopathological factor in the development of cerebral atrophy in hemodialysis patients. Nephron 1999; 81: 17-24
6. Zager PG, Nikolic J, Brown RH, Campbell MA, Hunt WC, Peterson D, Van Stone J, Levey A, Meyer KB, Klag MJ, Johnson HK, Clark E, Sadler JH, Teredesai P. “U” shaped curve association of blood pressure and mortality in hemodialysis patients. Kidney Int 1998; 54: 561-569
7. Port FK, Hulbert-Shearon TE, Wolfe RA, Bloembergen WE, Golper TA, Agodoa LYC, Young EW. Predialysis blood pressure and mortality risk in a national sample of maintenance hemodialysis patients. Am J Kidney Dis 1999; 33: 507-51
8. Zochalli C, Mallamaci F, Tripepi G, Benedetto FA, Cottini E, Giacone G, Malatino L. Prediction of left ventricular geometry by clinic, pre-dialysis and 24-hour ambulatory blood pressure monitoring in hemodialysis patients; CREED investigators. J Hypertens 1999; 17: 1751-1758
9. Salem MM, Bower J. Hypertension in hemodialysis population: any relation to one-year survival? Am J Kid Dis 28(5): 1996; 737-740
10. Goodkin DA, Bragg-Gresham JL, Loenig KG, Wolfe RA, Akiba T, Andreucci VE, Saito A, Rayner HC, Kurokawa K, Poert FK, Held PJ, Young WE. Association of comorbid conditions and mortality in hemodialysis patients in Europe, Japan, and the United States: the dialysis outcomes and practice patterns study (DOPPS). J Am Soc Nephrol 2003; 14: 3270-3277
11. Mazzuchi N, Carbonell E, Fernandez-Cean J. Importance of blood pressure control in hemodialysis survival. Kidney Int 2000; 58: 2147-2154
12. Degoulet P, Reach I, Rozenbaum W, Aime F, Devries C, Berger C, Rojas P, Jacobs C, Legrain M. Programme dialyse-informatique VI. Survie et facteurs de risqué. J Urol Nephrol 1979; 85: 909-962
13. Jindal K, Chan CT, Deziel C, Hirsch D, Soroka SD, Tonelli M, Culleton BF. Management of blood pressure in hemodialysis patients. J Am Soc Nephrol 2006; 17(suppl1): S8-S10
14. Rahman M, Griffin V, Kumar A, Manzoor F, Wright JT, Smith MC. A comparison of standardized versus “usual” blood pressure measurements in hemodialysis patients. Am J Kidney Dis 2002; 39: 1226-1230
15. Agarwal R, Lewis RR. Prediction of hypertension in chronic dialysis patients. Kidney Int 2001; 60: 1982-1989
16. Conlon PJ, Walshe JJ, Heinle SK, Minda S, Krucoff M, Schwab SJ: Predialysis systolic blood pressure and left ventricular mass in stable hemodialysis patients. J Am Soc Nephrol 1996; 7: 2658-2663
17. Cannella G, aoletti E, Ravera G, Cassottqna P, Araghi P, Mulas D, Pelosos G, Delfino R, Messa P. Inadequate diagnosis and therapy of arterial hypertension as causes of left ventricular hypertrophy in uremic dialysis patients. Kidney Int 58; 2000: 260-268
18. Liu M, Takahashi H, Morita Y, Maruyamaa S, Miauno M, Yuzawa Y, Watanabe M, Toriyama T, Kawahara H, Matsuo S. Non-dipping is a potent predictor of cardiovascular mortality and is associated with autonomic dysfunction in haemodialysis patients. Nephrol Dial Transplant 2003; 18: 563-569
19. Covic A, Goldsmith DJA, Covic M. Reduced blood pressure diurnal variability as a risk factor for progressive left ventricular dilatation in hemodialysis patients. Am J Kidney Dis 2000; 35(4): 617-623
20. Santos, SFF, Mendes RB, Santos CA, Dorigo D, Peixoto AJ. Profile of interdialytic blood pressure in hemodialysis patients. Am J Nephrol 2003: 23: 96-105
21. Amar J, Vernier I, Rossignol E, Bongard V, Arnaud C, Conte JJ, Slavador M, Chamontin B. Nocturnal blood pressure and 24-hour pulse pressure are potent indicators of mortality in hemodialysis patients. Kidney Int 57; 2000: 2485-2491
22. Cice G, Ferrara L, D’Andrea A, D’Isa S, Benedetto A, Cittadini A, Russo PE, Golino P, Calabro R. Carvedilol increases two-year survival in dialysis patients with dilated cardiomyopathy. J Am Coll Cardiol 2003; 41(9): 1438-1444)
23. Efrati Shai, Zaidenstein R, Dishy V, Beberashvili I, Sharist M, Averbukh Z, Golik A, Weissgarten J. ACE inhibitors and survival of hemodialysis patients. Am J Kidney Dis 2002; 40(5): 1023-1029
24. Kestenbaum B, Gillen DL, Sherrard DJ, Seliger S, Ball A, Stehman-Breen S. Calcium channel blocker use and mortality among patients with end stage renal disease. Kidney Int 2002; 61: 2157-2164
25. Foley RN, Herzog CA, Collins AJ. Blood pressure and long-term mortality in United States hemodialysis patients: USRDS waves 3 and 4 study. Kidney Int 2002; 62: 1784-1790
26. Klein IH, Ligtenberg G, Oey PL, Loomans HA, Blankestijn PJ. Enalapril and losartan reduce sympathetic hyperactivity in patients with chronic renal failure. J Am Soc Nephrol 2003; 4(2): 425-430
27. Gotch F: The current place of urea kinetic modeling with respect to different dialysis schedules. Nephrol Dial Transplant 1998; 13 (Suppl 6): 10-14
28. Woods JD, Port FK, Orzol S, Buoncristiani U, Young E, Wolfe RA, Held P. Clinical correlates of starting ‘daily’ hemodialysis. Kidney Int 1999; 55: 2467-2476.
29. Lindsay RM, Kortas C, Daily / Nocturnal Dialysis Study Group. Hemeral (Daily) hemodialysis. Adv Ren Replace Ther 2001; 8(4): 236-249.
30. Traeger J, Galland R, Arkouche W, Delawari E, Fouque D. Short daily hemodialysis: a four-year experience. Dial Transplant 2001; 30(2): 76-86
31. Galland R, Traeger J, Arkouche W, Delawari E, Fouque D: Short daily hemodialysis and nutritional status. Am J Kidney Dis 2001; 37(1): S95-S98.
32. Heidenheim AP, Muirhead N, Moist L, Lindsay RM. Patient quality of life on quotidian hemodialysis. Am J Kidney Disease 2003; 42(1): S34-S41
33. Ting G, White S, Lindsay RM. Requirements of an in-center daily hemodialysis program. Contrib Nephrol 2004; 145: 10-20
34. US Renal Data System USRDS 2002 Annual Data Report. National Institutes of Health, National Institute of Diabetes and Digestive and Kidney Diseases, Bethesda, MD, 2002
35. McCarthy JT, Moran J, Posen G, Leypoldt JK, Hull AR, Jaber BL, Correa-Rotter R. A time for rediscovery: chronic hemofiltration for end-stage renal disease. Semin Dial 2003; 16(3): 199-207
36. Quellhorst E, Hildebrand U, Solf A. Long-term morbidity: hemofiltration vs. hemodialysis. Contrib Nephol 1995; 113: 110-11.
37. Quellhorst EA, Schuenemann B, Mietzsch G. Long-term hemofiltration in ‘poor risk’ patients. Trans Am Soc Artif Internal Organs 1987; 33: 758-6.
38. Locatelli F, Marcelli D, Conte F, Limido A, Malberti F, Spotti D. Comparison of mortality in ESRD patients on convective and diffusive extracoporeal treatments. Kidney Int 1999, 55(1): 286-93.
39. Schaeffer J, Ehlerding G, Koch KM. Dialysis-related amyloidosis. Pathogenetic aspects and therapeutic considerations. Nephrology 1996; 2(suppl 1): S187-193.
40. Baldamus CA, Quellhorst E. Outcome of long-term hemofiltration. Kidney Int 1985; 28(17): S41-S46
41. Hulfer M, Asmus G, von Herrath D, Schaefer K. Hemodialysis or hemofiltration – the patients’ perspective. Blood Purif 1987; 5: 1-3
42. Lindsay RM, Kortas C, and the daily/nocturnal study group. Hemeral (daily) dialysis. Adv Renal Replace Ther 2001; 8(4): 236-249
43. Lindsay RM, Nesrallah G, Suri R, Garg A, Moist L. Is more frequent hemodialysis beneficial and what is the evidence? Curr Opin Nephrol Hypertens 2004; 13(6): 631-5
44. Pierratos A, McFarlane P, Chan CT. Quotidian dialysis-update 2005. Curr Opin Nephrol Hypertens 2005; 14(2): 119-24
45. Walsh M, Culleton B, Tonelli M, Manns B. A systematic review of the effect of nocturnal hemodialysis on blood pressure, left ventricular hypertrophy, anemia, mineral metabolism, and health related quality of life. Kidney Int 2005; 67(4): 1500-1508
46. Jaber BL, Zimmerman DL. Rationale and experience with short daily hemofiltration. Sem Dial 2004; 17(2); 146-150
47. Zimmerman DL. Hemofiltration as a treatment for end-stage renal disease. Hemodialysis Int 2004; 8: 183-187
48. Rabindranath KS, Strippoli GFM, Roderick P, Wallace SA, MacLeod AM, Daly C. Comparison of hemodialysis, hemofiltration, and acetate-free biofiltration for ESRD: systematic review. Am J Kidney Dis 2005; 45(3): 437-447
49. Parfrey PS, Harnett JD, Grififiths SM, Gault MH, Barre PE. Congestive heart failure in dialysis patients. Arch Intern Med 1988; 148: 1519-1525
50. Cox-Reijven PL, Looman JP, Soeters PB, van der Sande FM, Leunissen KM. Role of bioimpedance spectroscopy in assessment of body water compartments in hemodialysis patients. Am J Kidney Dis 2001; 38: 832-838
51. Cheriex EC, Leunissen KM, Janssen JH, Mooy JM, van Hooff JP. Echography of the inferior vena cava is a simple and reliable tool for estimation of ‘dry weight’ in haemodialysis patients. Nephrol Dial Transplant 1989; 4: 563-568
52. Koomans HS, Geers AB, Mees EJ. Plasma volume recovery after ultrafiltration in patients with chronic renal failure. Kidney Int 1984; 26: 848-854
53. Steuer RR, Leypoldt JK, Cheung AK, Senekjian HO, Conis JM. Reducing symptoms during hemodialysis by continuously monitoring the hematocrit. AM J Kidney Dis 1996; 27: 525-532
54. Steuer RR, Germain MJ, Leypoldt JK Cheung AK. Enhanced fluid removal guided by blood volume monitoring during chronic hemodialysis. Artif Organs 1998; 22: 627-632
55. Leunissen KM, Kouw PM, Looman JP, Cheriex EC, deVries PM, Donker AJ, van Hoof JP. New techniques to determine fluid status in hemodialyzed patients. Kidney Int 1993; 43(suppl41): S50-56, 1993
56. Katzarski KS, Charra B, Luik AJ, Nisell J, Filho JCD, Leypoldt JK, Leunissen KML. Laurent G, Bergstrom J. Fluid state and blood pressure control in patients treated with long and short haemodialysis. Nephrol Dial Transplant 14: 1999; 369-375
57. Zoccali C, Mallamaci F, Tripepi G, Benedetto FA, Cottini E, Giacone G, Malatino L: Prediction of left ventricular geometry by clinic, pre-dialysis and 24 hour ambulatory BP monitoring in haemodialysis patients. J Hypertens 17; 1991: 355-360

Amendments to the protocol:

#1: Removed the necessity for self-care as this was not going to be feasible with the staff that were available to train patients

#2: Changed the blood pressure criteria (SBP > 140 and 1 antihypertensive medication or 3 antihypertensive medications regardless of SBP) as there were concerns about being able to recruit the required number of patients for the study. Serum catecholamines were added to support the microneurography data.

#3: A decision was made not to adjust the patients dialysis prescription if the Kt/V was >1.4 as there were concerns about clearance of other solutes (potassium, phosphate) and a reduction in time would limit fluid removal

#4: Due to problems with the hemofiltration delivery system recognized almost immediately; the comparator arm was switched to daily hemodialysis
